# Representational shifts made visible: movement away from the prototype in memory for hue

**DOI:** 10.3389/fpsyg.2014.00796

**Published:** 2014-07-31

**Authors:** Laura J. Kelly, Evan Heit

**Affiliations:** Cognitive and Information Sciences, University of California, MercedMerced, CA, USA

**Keywords:** representation, memory, categorization, labels, perception, color, typicality

## Abstract

In four experiments, a total of 205 participants studied individual color patches and were given an old-new recognition test after a brief retention interval (0.5 or 5.0 s). The pattern of hue sensitivity (*d*′) revealed hue memory shifting away from the prototype of the hue's basic color category. The shifts demonstrate that hue memory is influenced by categorization early in processing. The shifts did not depend on intentional categorization; the shifts were found even when participants made preference ratings at encoding rather than labeling judgments. Overall, we found that categorization and memory are deeply intertwined from perception onward. We discuss the impact of the results on theories of memory and categorization, including the effects of category labels on memory (e.g., Lupyan, 2008). We also put forward the hypothesis that atypical shifts in hue are related to atypical shifts that have previously observed in face recognition (Rhodes et al., 1987).

## Introduction

Memory, reasoning and categorization have traditionally been distinguished as separate topics and separate areas of research (Heit and Hayes, 2005; Heit et al., 2012; Hayes et al., 2014). It could be argued that categorization is either an automatic process as in categorical perception, where conscious reasoning is not recruited and any effect of categories on perception would appear to be due to the activation of categorical memory, or alternatively, categorization is an explicit process as in categorical decision tasks where a more deliberative process of reasoning may be at work. But the dichotomies of implicit vs. explicit and memory-based vs. reasoning-based categorization are too extreme; instead, a continuum is likely to exist. We suggest that these cognitive activities are intimately intertwined. As Churchland (1981) pointed out, the terms we have from folk psychology, the ways we culturally divided cognitive processes prior to having scientific evidence to inform those divisions, are not necessarily sensible. As research advances in psychological and neurological understandings of cognitive processes, these traditional terms and divisions need to be broken down. The memory, reasoning and categorization distinctions are losing their usefulness as separate constructs due to the likelihood of common underlying mechanisms.

In this paper, we will be looking at a task that involves both memory and categorization. Experimental participants either label hues with basic color categories or make preference judgments about the hues. Then, memory for these hues is tested immediately. Participants have memory of categories and through categorization bring that memory to bear on newly formed encodings. In the way categorization and memory are often discussed, categorization is the act of applying knowledge while memory is the substance of that knowledge. Yet using memory edits memory itself, as has been shown with memory reconsolidation (Nadel et al., 2012) and retrieval induced forgetting (Anderson et al., 1994). Memory and categorization cannot be treated as fully distinct cognitive topics but are interdependent.

The distinction between perception and memory is also a vague and possibly false distinction. Perception is the transduction of light, sound waves, chemicals, pressure, and heat into electrical signals in the nervous system. Memory refers to the storage of that information. Milliseconds after a stimulus has been experienced, researchers consider it remembered in iconic memory, some of which passes on to working memory and possibly to long-term memory. There has been a debate about how far top-down conceptual knowledge can impact perception with some arguing that perception is cognitively impenetrable (Pylyshyn, 1999) and others arguing that cognitive expectations affect perception very early in processing (Churchland, 1988; Hsieh et al., 2010).

One of the main phenomena of interest in the cognitive penetrability debate is categorical perception, where categorical knowledge affects how people perceive the surrounding world. Categorical perception has been examined in many domains including phoneme perception (Liberman et al., 1957), faces (Levin and Beale, 2000), and color (Winawer et al., 2007), (see Goldstone and Hendrickson, 2010 for a full review). In categorical perception, there is no deliberative reasoning—categorization is implicit and automatic, having an effect without people needing to actively decide on a category. Here, categorization appears to be based on implicit memory of frequent categorizations.

Categorical perception has been explained diversely: as a pull toward the prototype (Lupyan, 2008), a truncation at the boundaries of a category (Huttenlocher et al., 1991), or an expanding of perceptual space (Goldstone, 1994, 1998). None of these accounts of categories on perception would explain the novel result we present here: With rapid presentation and test of hues there is an atypical bias—a push away from the prototype, a pull toward the boundary, or a seemingly incompatible change of perceptual space. While this result is novel for hue memory, a similar effect has been observed in immediate recognition of exaggerated faces (Rhodes et al., 1987). During perception, people appear to bring categories to bear on the content of perception but the influence is not uniformly one of attraction toward the prototype.

Our own investigation was spurred by the argument that labels affect the memory of perception when the labels coincide with perception (Lupyan, 2008). Specifically, for an effect that was metaphorically referred to as a representational shift, it was claimed that labels cause memory traces to be prototypically shifted from the raw percept by exerting a top-down influence of the labeled category on the perceived item. The label activates the category prototype, which interacts in real time with the bottom up perception resulting in a mixed encoded memory trace. Specifically, these experiments looked at whether there was an advantage to remembering objects that were labeled or judged in terms of preference (liking). The participants either labeled object categories including chairs, lamps, and tables (two categories per experiment) or made a like/dislike preference judgment in alternating blocks during study. Participants only saw the objects for 300 ms and had 700 ms to respond to discourage labeling in the preference judgment trials. After all study trials, participants were then tested on their memory for the items using the original objects as well as a matched lure for each original item in a new/old recognition task. Participants less accurately remembered previously seen items if they had been categorically labeled, which was taken as evidence that the representation of the labeled objects was shifted—it no longer matched up to the originally perceived item. Other researchers (Richler et al., 2011; Blanco and Gureckis, 2013) have taken issue with this interpretation in terms of representational shift, instead suggesting that perceived items are remembered better because preference judgments require a greater depth of processing than category labeling. They introduced non-labeling conditions such as chair orientation (Blanco and Gureckis, 2013) and screen position (Richler et al., 2011) that only require superficial processing of the objects. These conditions performed similarly to the category labeling condition introduced by Lupyan (2008). To these researchers, the strength of the memory accounts for the differences in recognition memory.

The controversial claim from Lupyan (2008) that there are prototypical representational shifts has not been demonstrated directly. Only a decrease in accurate recognition of previously seen items has been shown, which could mean a shift toward the prototype, away from the prototype, or simple non-directional forgetting. Hence, previous research on representational shifts has not provided clear evidence that representations have shifted, much less in what direction they have shifted. To get at this question we will present a paradigm that is a conceptual replication of Lupyan (2008) using the same judgment conditions at target presentation, category labeling and preference judgments, and using a similar memory test, same/different judgments rather than new/old recognition judgments. The main differences are in stimuli and timing. We present the targets as well as four matched lures varying systematically in category typicality and distance from the target. These stimuli will allow us to quantify the direction and magnitude of any representational shifts that occur. If a shift is in the typical direction as predicted by Lupyan (2008), the new array of test stimuli will allow it to be seen.

Previous work on representational shifts has examined memory for objects such as lamps and chairs. In our own work, we focus on color space, which is more quantifiable and better-defined than object space. Color is a continuous uniform physical space made up of different wavelengths of light. Color is also a rich psychological space that is divided into superordinate, basic, and subordinate categories. There are focal or prototypical colors within categories as well as boundaries where one category meets the next that are shared amongst speakers of the same language, and to some extent across languages (Berlin and Kay, 1969; Regier and Kay, 2009). As such, color space is a fertile testing ground for examining how categorical knowledge distorts basic perception.

Taking account of the psychological landscape of the color domain and people's ability to detect fine alterations from one color stimulus to the next, we were able to directly test the color that has been encoded through a recognition test, and how different, if at all different, the encoded color is from the originally presented color. By moving from object space to hue space, we constrain the potential directions of memory shifts toward or away from the prototype. By testing memory of the target as well as four matched lures differing in distance and direction in hue space relative to the prototype, we have the opportunity to measure the sensitivity (*d*′) of hue memory at different locations relative to the target. Sensitivity serves as a measure of confusability and strength of confidence in having seen something at the point in hue space. Where *d*′ is high, people can reject lures that they have not seen. Where *d*′ is low, this means that lure items nonetheless seem relatively familiar, as if there is a false memory representation at that point in hue space. Moreover, if *d*′ is lower in one direction, relative to the prototype, compared to the other direction, this implies that the representation in memory has shifted directionally. Our paradigm allows us to see the shift as well as quantify its direction and strength.

Additionally, the representational shift hypothesis focuses on encoding. However, the paradigm used in the original paper (Lupyan, 2008) as well as the versions of the paradigm used in subsequent work (Richler et al., 2011, 2013; Blanco and Gureckis, 2013) have an extended study phase presenting all items twice prior to a test phase of all items resulting in a delay of minutes between presentation and test. This format does not isolate effects down to the time of encoding. Our paradigm focuses on immediate memory to more closely address encoding. We use a same/different judgment as the memory test either 500 ms after target presentation (Experiments 1A and B) or 5000 ms after target presentation (Experiment 2A and B). This prevents interference of other hues on the representation of the key item between study and test.

We now present four experiments, two main experiments and two direct replications. Experiment 1A was designed to test the memory for a color soon after encoding. The delay between original presentation and the same/different judgment was 500 ms. We found an atypical shift—unexpected based on previous research which had suggested that the shift would be toward the prototype—with no difference between judgment conditions. In Experiment 2A, the delay was increased to 5000 ms to test if the predicted prototypical shift could be observed at a longer delay and if there was an effect of judgment condition that developed over time. The atypical shift and lack of judgment condition effect were reproduced. Due to some participants being excluded from Experiments 1A and 2A as well as the unexpected direction of the representational shift, we conducted direct replications of both experiments with higher power, in Experiments 1B and 2B.

## Experiment 1A

In a conceptual replication of Lupyan (2008), we presented participants with hues to judge either by category or by preference. In a departure from the previous paradigm that had separate study and test phases, test occurred immediately after study within a trial. Given that the representational shift hypothesis is one of shift at encoding, shifts should be immediately detectable. Additionally, rather than having one matched lure for each studied item, there were four lures spanning both potential directions of movement relative to the prototype and two distances in hue space. Using sensitivity (*d*′) as the dependent measure, we determined whether memory shifted at all, if it shifted toward the prototype or away from it, and the approximate distance of the shift in hue space. In particular, lower *d*′ values indicate a higher false-alarm rate to lures. So, for example, if representations shift toward the prototype, there will be greater likelihood of false-alarming to typical lures compared to atypical lures, and *d*′ will be lower for typical items than for atypical items.

### Methods

#### Participants

Thirty-six students at the University of California, Merced participated in these experiments for course credit. All participants reported normal vision and normal color vision. Their color vision was tested using the CITY colorblindness test (City University, 2002) following the main experiment. The research was approved by the University of California, Merced Institutional Review Board and verbal consent was obtained from each participant.

#### Materials

The color stimuli were calculated in CIE L^*^CH color space then translated to CIE L^*^ab color space. The stimuli were from two color categories, red and green. Focal colors, treated as the category prototypes, were obtained from Sturges and Whitfield (1995). Saturation and brightness were held constant at the focal saturation and focal brightness. Within these color categories, four target colors were selected for a total of eight target colors across the two categories. All target hues were of similar typicality relative to the prototypes though explicit typicality measures were not collected. The targets were neither extremely typical nor atypical of their color category. From each of the target colors, four variants were created, two closer to the prototype and two further away from the prototype. These variants served as the recognition test lures. The hue distance between each hue in the set of 5 test hues, the target and four lures, was equal. The hue distances were normalized for the different color spaces with green encompassing a larger number of degrees than red. All variants within a set did not cross the prototype or the color category boundaries. The calculated colors can be found in Supplementary Materials: Appendix A.

Dell Ultrasharp U2410 monitors were used to display the stimuli and the color calibration profiles were created using a X-rite i1 Display Pro color calibrator. The stimuli were created using Adobe Photoshop to convert the calculated colors to a RGB device specific color profile for each monitor, resulting in uniform presentation across the three monitors. Using a photometer, the experimental cubicles were found to have similar intensities of light.

#### Procedure

There were two target judgment conditions. Participants chose between the basic color categories, green and red, for the categorical judgment and between like and dislike for the preference judgment. The categorical judgment response keys were counter-balanced across participants whereas the like/dislike response keys were in left to right order as it is a natural mapping. The second judgment of each trial was a same/different judgment. The participant was to judge whether the second hue presented during the trial matched the first hue that had elicited the category or preference judgment.

Each trial consisted of a fixation cross (1500 ms), the target hue (300 ms), a question mark eliciting a button push judgment (up to 700 ms), a blank screen (500 ms) and a response screen with a test hue also eliciting a button push judgment (up to 4000 ms). The participant's response immediately ended the response-eliciting screens. The trials were portioned into blocks of 80 trials consisting of all 8 target colors being paired with their 5 test hues (the original hue and the 4 lures) for 2 trials each. Each block had one type of judgment (categorical or preference) being the response to the question mark. There were 4 blocks, alternating between the judgment types. The order of the blocks was counter-balanced across participants.

Prior to the main trials, participants were trained on 6 yellow and purple stimuli trials, then completed 2 short blocks of 10 trials, one block of category judgments and one block of preference judgments to allow participants to get into the rhythm of responding quickly before the key trials began. These short blocks contained the red and green stimuli and were not indicated to be practice trials to the participants.

### Results and discussion

We excluded 12 participants, 8 for low color naming accuracy (<80% correct) in spite of color vision screening, as well as 4 for failing to follow instructions. Failing to follow instructions in this and subsequent experiments included a very high rate of “like” judgments >90%, a low response rate at either the judgment or test portion of a trial (<80%), or always responding with “same” at test. Including these participants does not change the pattern of results. Here, we report results based on 24 remaining participants. In this and subsequent experiments, individual trials were excluded if categorization at study was incorrect or participants did not respond at both the study and test portions of a trial.

The analyses relied on the *d*′ measure of sensitivity used in signal detection theory (Stanislaw and Todorov, 1999). The *d*′ measure has been used in recent studies of representational shift (Richler et al., 2011, 2013; Blanco and Gureckis, 2013) though false alarm rates alone were used in the original paper (Lupyan, 2008). Compared to analyses based on raw scores such as false alarm rates, *d*′ not only takes account of variations in hit rate but has the advantage of being a better match for the underlying Gaussian nature of recognition data (see Macmillan and Creelman, 2004, for a general overview, and Heit and Rotello, 2014, for a more recent discussion). For the analyses, we calculated two overall hit rates per participant one for each condition and used these along with the four lure false alarm rates per condition to calculate *d*′ values. By using *d*′ rather than raw false alarm rates, we are controlling for the general response rate of an individual in a condition in addition to calculating how well they can differentially respond to the target vs. the lures.

In this case, we had one set of test items that were the same as the originally presented item. Same judgments on these items were considered hits and different judgments were considered misses. We also had 4 sets of items that were different from the original hue varying in hue space distance (1 step or 2 steps) and in direction of typicality (more typical or more atypical of the color category). Same judgments in response to these items were false alarms and different judgments were correct rejections. We calculated *d*′ by subtracting the z-score of the proportion of false alarms from the z-score of the proportion of hits. In the case of proportions of 0 and 1, z-scores cannot be calculated due to the normal curve expanding to infinity at its tails. We used the standard correction of including or excluding half a hit or half a false alarm where appropriate (Snodgrass and Corwin, 1988). The hit and false alarm rates for all experiments are reported in Supplementary Materials: Appendix B. The *d*′ measure was calculated for each of the four levels of the test hue variations and for each of the two judgment conditions by subject.

A significantly lower level of sensitivity, namely a lower *d*′, was taken to be evidence of the direction of a shift. Lower *d*′ corresponds to more false alarms or more non-targets confused to be the same as the target. So, for example, if there is lower *d*′ as a result of more false alarms for prototypical items, this suggests a prototypical shift—the memory traces are more similar to the more typical test items than the less typical items. Likewise, lower *d*′ for atypical items suggests an atypical shift. No difference, or a symmetrical sensitivity, would imply that memory does not shift relative to the category typicality gradient. The representational shift hypothesis (Lupyan, 2008) suggests that there should be an interaction of typicality and condition with a lower *d*′ for the more typical lures than the atypical lures only in the category labeling condition. There should be no typicality effect, or at least a smaller effect, for the preference condition. The depth of processing account (Richler et al., 2011; Blanco and Gureckis, 2013) predicts a main effect of condition with no typicality effect; the sensitivities should be symmetrical. The key prediction of this account is more accurate memory, a higher *d*′, for items in the preference condition which is suggested to be more deeply processed than the items in the category labeling condition.

**Figure 1:**
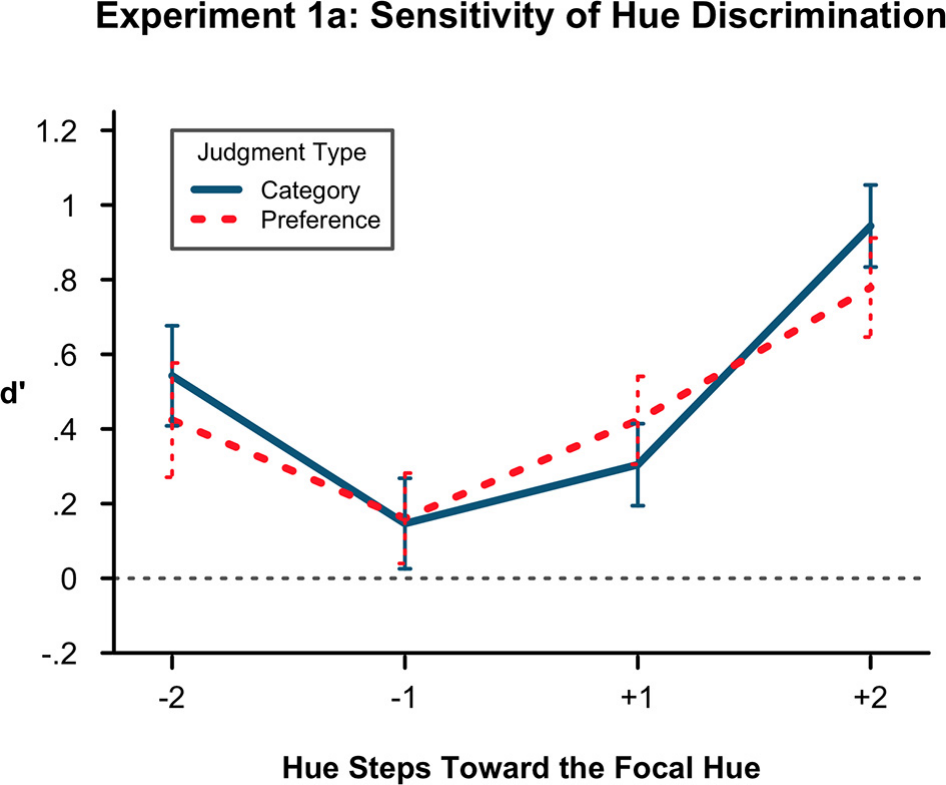
**Sensitivity of hue discrimination in Experiment 1A**. Lower *d*′ values indicate more confusion of hues at a particular level of the hue factors, here denoted with sign for toward (+) or away (−) from the prototype and with number (1, 2) for steps distant from the original hue. Here, *d*′ is lower in the atypical (−) direction than in the prototypical. It is also lower at 1 step removed from the originally presented hue than at 2 steps. There is no difference by condition. Error bars represent the standard error of the means.

To test whether there was a difference in *d*′ by condition (color vs. preference judgment), distance from the original hue (1 step vs. 2 steps in hue space) or by direction of typicality (typical vs. atypical), we ran a 2 × 2 × 2 ANOVA. The results can be seen in Figure 1. There was no effect of condition, with labeling the category or making a preference judgment not differentially affecting hue sensitivity. There was a main effect of distance, *F*_(1, 23)_ = 29.59, *p* < 0.001, η^2^ = 0.184. The distance of 2 units had a *d*′ mean of 0.672 while the *d*′ mean of the distance of one unit was 0.259, indicating that there was less sensitivity to a hue change when the test hue was closer to the original hue as one would expect. This finding indicates that the hues that are less different in color space are less detectable. Therefore, any shift that has taken place with the color hues is subtle and within a few degrees of hue space.

The key finding was a main effect of typicality, *F*_(1, 23)_ = 12.92, *p* < 0.01, η^2^ = 0.100. The *d*′ mean of more typical test hues was 0.612 and the *d*′ mean of hues less typical of the color category was 0.318, indicating that participants were less sensitive to changes in hue if the hue was atypical of the color category. In other words, participants were more likely to false-alarm to atypical test items than to typical test items. Based on prior theoretical work, the prediction was actually the opposite, that there would be less sensitivity and therefore a representational shift in the typical direction.

Additionally, there were two marginal components of the ANOVA, an interaction of condition and distance [*F*_(1,23)_ = 3.11, *p* = 0.091] and an interaction of distance and typicality [*F*_(1.23)_ = 3.11, *p* = 0.112]. Due to the unexpected result, the marginal findings, as well as the number of participants excluded resulting in a small final sample size, we view conducting a direct replication as important to having confidence in our results (see Cesario, 2014 for discussion of the importance of direct replication).

## Experiment 1B

### Methods

Sixty-seven students participated using the same criteria as in Experiment 1A. All materials and procedures were the same.

### Results and discussion

We excluded 22 participants, 10 for low color naming accuracy (<80% correct) in spite of color vision screening, as well as 12 for failing to follow instructions. Including these participants does not change the pattern of results. Here we report findings based on 45 remaining participants.

**Figure 2:**
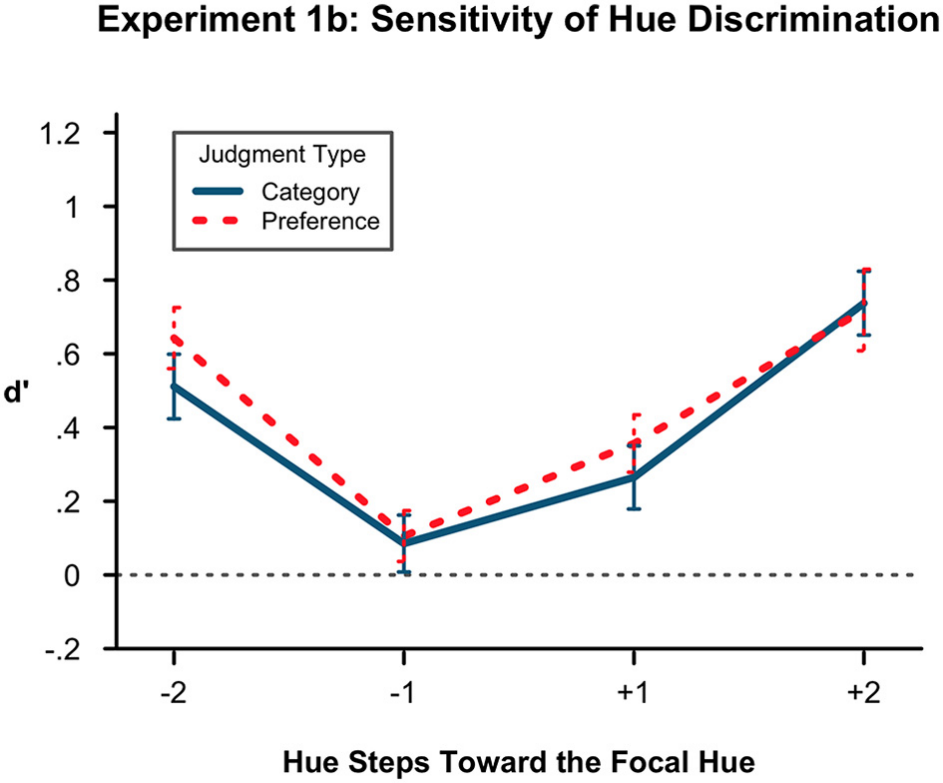
**Sensitivity of hue discrimination in Experiment 1B**. Lower *d*′ values indicate more confusion of hues at a particular level of the hue factors, here denoted with sign for toward (+) or away (−) from the prototype and with number (1, 2) for steps distant from the original hue. Here, *d*′ is again lower in the atypical (−) direction than in the prototypical direction. It is also lower at 1 step removed from the originally presented hue than at 2 steps. There is no difference by condition. Error bars represent the standard error of the means.

The results are shown in Figure 2. Conducting the same 2 × 2 × 2 ANOVA on the *d*′ scores, there was again no effect of condition. We replicated the significant main effect of distance, *F*_(1, 44)_ = 83.58, *p* < 0.001, η^2^ = 0.242 indicating that again it was easier to distinguish items further from the original with the *d*′ mean of 2 units being further from zero at 0.652 than the mean of the 1 unit hue distance items at 0.203. We also replicated our typicality main effect, *F*_(1, 44)_ = 10.39, *p* < 0.01, η^2^ = 0.048, with the atypical direction mean of 0.336 and the typical direction mean of 0.519. Again, participants were less sensitive to changes in hue if the change was away from the category prototype. None of the interactions were significant including the previously marginal results.

With this replication we can have more confidence in concluding that the representational shift is occurring in the atypical direction.

## Experiment 2A

The representational shifts at a half second delay between presentation and test in Experiments 1A and B were in the atypical direction. Lupyan (2008) had argued that labeling should have an effect of increasing the typicality of a representation at encoding. Here, we increased the delay between presentation and test to 5 s to see if a labeling effect or a reversal in the direction of the shift emerged with more processing time.

### Methods

Forty students were recruited as in the other experiments. The materials and procedure were the same as in Experiments 1A and B with the exception of increasing the delay of the blank screen between the original hue presentation and the test hue presentation from 500 ms to 5000 ms.

### Results and discussion

We excluded 21 participants for low color naming accuracy (<80% correct) in spite of color vision screening. Including these participants does not change the pattern of results. Here, we report results based on 19 remaining participants.

**Figure 3:**
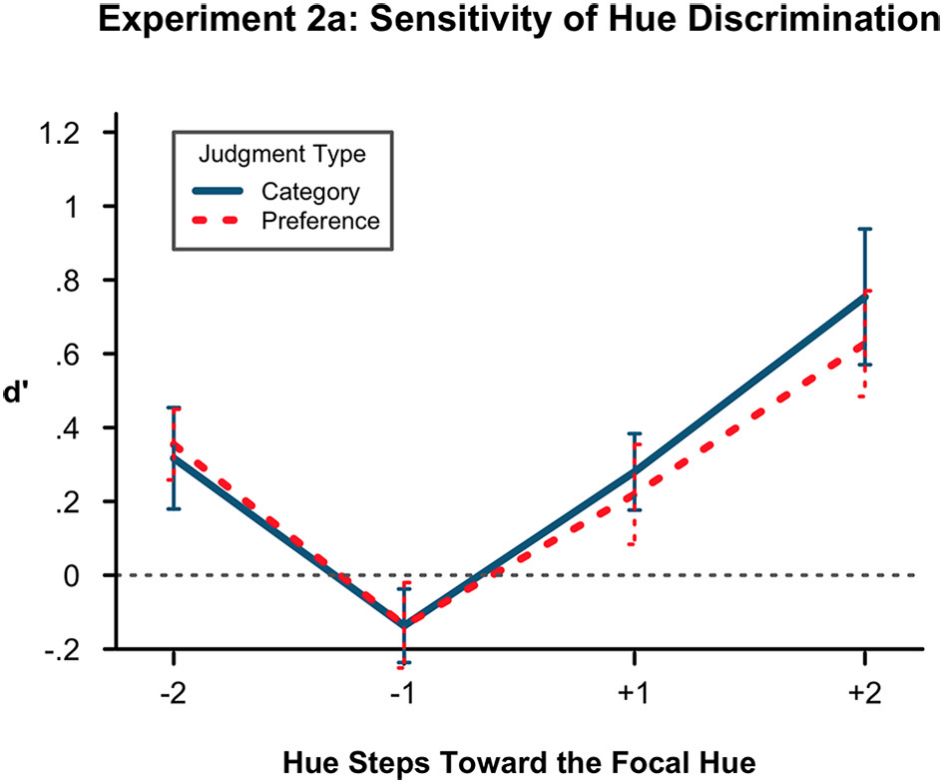
**Sensitivity of hue discrimination in Experiment 2A**. Lower *d*′ values indicate more confusion of hues at a particular level of the hue factors, here denoted with sign for toward (+) or away (−) from the prototype and with number (1, 2) for steps distant from the original hue. Here, *d*′ is again lower in the atypical (−) direction than in the prototypical direction. It is also lower at 1 step removed from the originally presented hue than at 2 steps. There is no difference by condition. Error bars represent the standard error of the means.

We conducted a 2(color vs. preference judgment) × 2(1 hue step vs. 2 hue steps) × 2(typical vs. atypical direction) ANOVA on *d*′ as in the previous experiments. Figure 3 shows the *d*′ means and error at each level of the ANOVA. There was no effect of condition. We found a main effect of distance [*F*_(1,18)_ = 54.18, *p* < 0.001, η^2^ = 0.297] with the 2 units of hue distance being more detectable (mean = 0.513) than the 1 hue step (mean = 0.057). We also again found a main effect of typicality [*F*_(1, 18)_ = 21.99, *p* < 0.001, η^2^ = 0.204) with more atypical hues being less detectable (mean = 0.1) than more typical hues (mean = 0.47). No interactions were significant.

As in Experiments 1A and B, there was no effect of condition and a significantly lower sensitivity in the atypical direction, pointing again to a shift away from the prototype. However, there was again a relatively high number of exclusions resulting in a low final sample size. We again conducted a direct replication.

## Experiment 2B

### Methods

We recruited 62 participants using the same criteria as the previous experiments. All materials and procedures were the same as in Experiment 2A.

### Results and discussion

We excluded 14 participants, 7 for low color naming accuracy (<80% correct) in spite of color vision screening and an additional 7 for failing to follow instructions. Including these participants does not change the pattern of results. Here, we report results based on 46 remaining participants.

**Figure 4:**
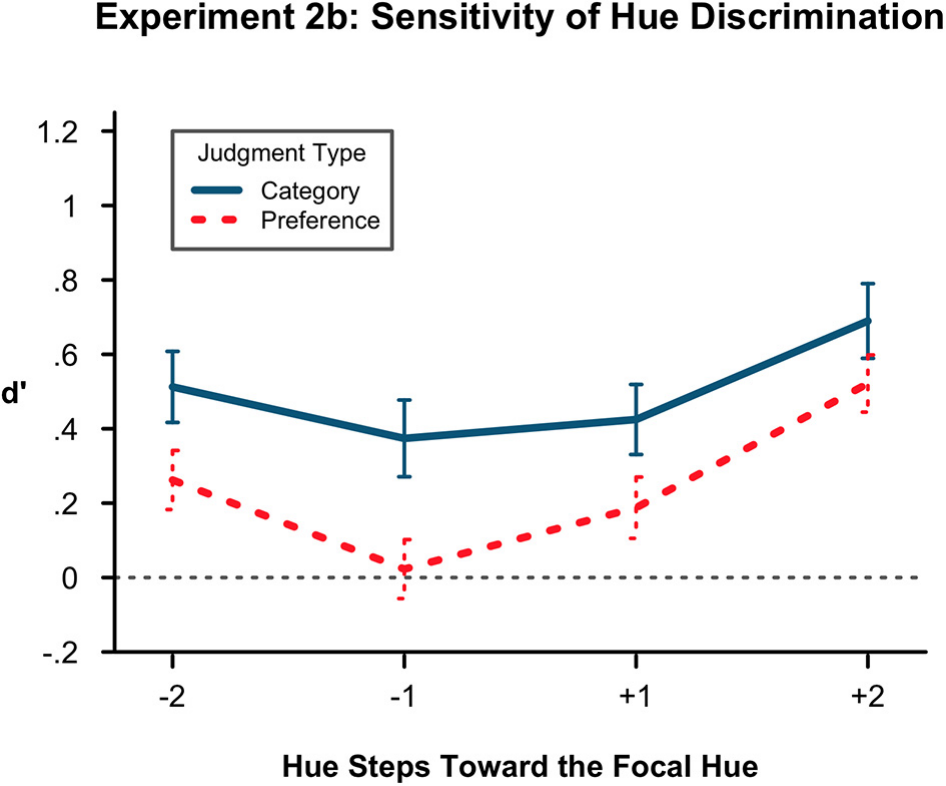
**Sensitivity of hue discrimination in Experiment 2B**. Lower *d*′ values indicate more confusion of hues at a particular level of the hue factors, here denoted with sign for toward (+) or away (−) from the prototype and with number (1, 2) for steps distant from the original hue. Here, *d*′ is again lower in the atypical (−) direction than in the prototypical. It is also lower at 1 step removed from the originally presented hue than at 2 steps. There is a difference by condition with less sensitivity in hues that were preference-judged over hues that were category-labeled. Error bars represent the standard error of the means.

We again conducted the same 2 × 2 × 2 ANOVA on the *d*′ measurements (Figure 4). There was a main effect of judgment condition in this experiment unlike the 3 others, *F*_(1, 45)_ = 6.26, *p* < 0.05, η^2^ = 0.067. Preference-judged items (mean = 0.249) were less detectably different from the original than color judged items (mean = 0.500). In other words, there were more false alarms, and more shifting, for preference judgments than for labeling judgments. There was also a main effect of distance [*F*_(1, 45)_ = 30.60,*p* < 0.001, η^2^ = 0.103], replicating the finding that items 2 hue steps distant from the original (mean = 0.496) are more detectable than items 1 step distant (mean = 0.252). We also replicated our previous main effect of typicality [*F*_(1, 45)_ = 7.48, *p* < 0.01, η^2^ = 0.043] with more atypical items being less detectable (mean = 0.293) than more typical items (mean = 0.456). Again, no interactions were significant.

Whereas *d*′ is a direct measure of discrimination, for completeness we conducted *post-hoc* analyses of the raw false alarm rates using the same 2 × 2 × 2 ANOVA. (See also Supplementary Materials: Appendix B). In all of the previous experiments reported here, the pattern of results was consistent with the ANOVA conducted on *d*′ scores. In this experiment, while the false alarm rates of the foils continued to show the typicality effect, *F*_(1, 45)_ = 8.688, *p* < 0.005, and the distance effect, *F*_(1, 45)_ = 31.1, *p* < 0.001, the main effect of condition was not significant when false alarm rate was the dependent variable, *F*_(1,45)_ = 0.006, *p* = 0.939. The main effect of condition in the *d*′ analysis was driven by taking into account the participants' high labeling condition hit rates.

Overall, we replicated the direction of the shift. That is, extending the time between study and test from half a second to 5 s did not change the atypical nature of the representational shift. Here we observed a difference between conditions for the first time. Category-labeled hues were more distinguishable from the original than those that were preference-judged. We hesitate to draw too strong a conclusion from this difference since it was only found in one of the four experiments and the false alarm analysis implied that the condition effect depends on the hit rate rather than the false alarm rate. Interestingly however, with only a main effect and no significant interaction, the category labeling increased sensitivity to hue differences overall, rather than in a particular direction toward or away from the prototype.

## General discussion

In our experiments, we found less sensitivity to differences between a studied hue and an unstudied test hue that is less typical of the category. In other words, participants were more likely to false-alarm to atypical items than to typical items. We take this as evidence that there are representational shifts and that they are away from the prototype. Additionally, there may be a judgment condition effect that emerges over time, with category labeled hues being more easily detected as different from the original hue. There is no interaction of the condition and the typicality direction indicating that while category labeled hues might be more detectable, it is not due to shifting. Instead, we can speculate that labeling a color allows participants to reduce bias in either direction equally.

### Representational shifts, depth of processing, or transfer appropriate processing

The representational shift hypothesis (Lupyan, 2008) predicted that (1) there are representational shifts, (2) the shifts happen at encoding, (3) shifts are in the prototypical direction, and (4) category-labeled items are more strongly shifted. Our paradigm was able to show that there are representational shifts and that they occur very quickly, possibly at encoding. However, we found atypical shifts instead of the predicted prototypical shifts. Additionally, we found shifts for both category labeling judgments and preference judgments, indeed in Experiment 2B with stronger shifts in the preference condition. Given these results, we question whether the representational shift hypothesis as detailed by Lupyan is the appropriate explanation here.

Depth of processing (Craik and Tulving, 1975; Richler et al., 2011; Blanco and Gureckis, 2013) does not fit the present results either. If preference judgments require greater depth of processing and depth of processing leads to better memory, then preference judgments should lead to more sensitivity to hue changes. In fact, we found that preference judged hues led to either indistinguishable sensitivity or less sensitivity than category-labeled hues. Therefore, the depth of processing account of the representational shifts is not satisfactory either.

Instead, we point to transfer appropriate processing (Morris et al., 1977) as a potential framework in which to understand the results. In transfer appropriate processing, relevant details to the task at hand are processed with more depth than details that are less appropriate at the time of encoding. Perhaps preference judgments have an inherently greater depth of processing compared to basic categorization, but the content of that depth is not necessarily what is needed for greater sensitivity in the present task. Directing processing into comparison of the hue against preferences and making a valence judgment may distract from the encoding of the exact hue while color labeling concentrates processing on the appropriate aspect of the hues for greater sensitivity. Rather than greater raw processing, the right kind of processing leads to more exact memory.

### Atypical shifts

Counterintuitively, the representational shifts at a rapid test pace were in the atypical direction. Previous research on categorical knowledge effects on memory mostly suggests that if memory is altered systematically from the original percepts it should be in a prototypical direction (e.g., Heit, 1997). Categories serve to generalize our knowledge and to highlight similarity among distinct exemplars. What purpose could be served by atypical shifts? While we will not claim to have a final answer to this question, we speculate that it is related to perceptual expertise processes.

A domain where a similar atypical representational shift has been found is in recognition of faces. This has been called a distinctiveness effect (Rhodes et al., 1987). Participants were faster to recognize exaggerated faces over the original facial proportions that were in turn recognized faster than more generic versions of the faces. The authors argued the most distinctive features of a face are what are encoded into memory with the more generic portions not encoded as strongly. Gist memory (Reyna and Brainerd, 1995) can be used to fill in the representation. When a person then goes to use the encoding to recognize a face, the exaggerated face matches the distinctive features better than the true face.

Hue is much less complex than faces—just a single dimension of a single feature—and yet, we found a similar bias away from the prototype. Rather than encode the one feature veridically, participants appear to have encoded a shifted hue. Perhaps the mechanism that underlies the caricature effect is a magnification of the atypical effect we observed through multiple features all moving atypically. While the details of how this one feature case relates to the more complex case of faces is unclear, our results call into question the explanation that we simply encode the distinctive features of a face as they are without the more generic aspects to achieve an exaggerated encoding.

There has been a long debate over whether faces are in some way special in object processing (Farah, 1996; Kanwisher et al., 1997; Toveé, 1998; Gauthier and Logothetis, 2000; McKone et al., 2007). The majority of humans, those without a specific deficit called prosopagnosia, are considered to be experts at facial recognition. On the domain general, expertise explanation of face processing effects, visual object domains other than faces such as cars and birds can be processed in similar ways with experience (Gauthier et al., 2000). Bukach et al. (2006) advocated the use of an expertise framework to understand category specialization. Colors are generally associated with a basic level of categorization (Berlin and Kay, 1969; Rosch, 1975). When forced by a task to make fine subordinate distinctions, a different strategy appears to emerge. Movement in the atypical direction in our experiments was relative to basic categories. Perhaps the fine-grained categorization process is overcompensating for a more natural generalization and homogenization process that occurs when the participant is functioning at the basic category level. The detailed memory of hue demanded of participants in this task was not a typical activity. But for faces, fine-grained distinctions are a basic need. This may indicate that expert processing techniques can be flexibly recruited in real-time to a task and do not depend exclusively on trained distinction making within a domain.

### Online role of labels

While the prototypical shifts predicted by the representational shift hypothesis were not found and the mechanism underlying the shifts proposed by that hypothesis was not supported, the larger framework of the label-feedback hypothesis (Lupyan, 2012) is not something we are looking to challenge. In the label-feedback hypothesis, language is a pervasive online influence on cognition in the tradition of Whorf (1956). Language is an inherent part of the complex multidimensional system of the normal human adult mind, not something that is switched on and off depending on the task. Instead, labels serve to up-regulate the influence of linguistic knowledge online while verbal interference down-regulates that influence.

One interpretation of the current results would be compatible with the label-feedback hypothesis. Namely, the influence of the labels on memory occurs online and serves to increase the sensitivity of an individual's ability to detect change in the labeled category. In this account, the up-regulation of language's influence in the labeled case allows processing to focus in on the hue resulting in more accurate memory. Sloutsky (2003) discussed the role labels have in directing attention during category learning to relevant similar features among items in a labeled category. Extending the logic from learning to use of learned categories, if labels are features of the category, invoking them will draw attention to the dimension(s) on which the category similarity and distinctions are judged. While labeling did not have the effect of pulling items toward the category prototype in this task, labeling could have a more general modulating influence on encoding.

An alternative explanation could be somewhat consistent with the label-feedback hypothesis but from the opposite direction. The basic categories of color could be essentially automatic (Grill-Spector and Kanwisher, 2005) having language's influence close to ceiling. The preference judgments may serve to distract processing from reaching the level of depth it naturally would regardless of the color label because preference valence needs to be the focus of directed attention (Simons, 2000). Preference judgments would be down-regulating the influence of linguistic category knowledge.

We are agnostic given the present evidence whether labeling has an added effect, the preference judgments have a distracting one, or some combination of the two is at play. Disambiguating the competing interpretations would be an interesting direction for further research. Either way, attention appears to be directed at the relevant dimension for the memory test when colors are labeled while attention is on a different dimension when preferences are being elicited.

### Categorical perception

Our results can also speak to recent developments in the categorical perception literature. Categorical perception is the effect of enhanced discrimination performance when the items being discriminated cross category boundaries. This has been attributed to changes in perception (Harnad, 1987), particularly the enhanced distinctiveness of learned category differences (Goldstone, 1994). Roberson and colleagues (Roberson et al., 2009; Kikutani et al., 2010) proposed a different account suggesting that category labels play a crucial role in categorical perception, with different labels facilitating greater accuracy and faster reaction times.

Hanley and Roberson subsequently updated their account. Conducting a reanalysis of a series of two alternative forced choice categorical perception tasks discriminating between colors or faces (Hanley and Roberson, 2011), they found an asymmetry among the within trials that are traditionally treated as a single condition. On trials where the target item was more typical of the category, or a better exemplar, compared to the foil, participants had a similar proportion correct to between category trials. On trials where the target item was more atypical of the category than the foil, a poor exemplar, participants performed much worse. These poor exemplar trials account for the overall categorical perception effect. Hanley and Roberson account for this finding through the relative reliability of labels applied to the items. If participants labeled a hue blue when the hue was on its own, the participant who remembers “blue” rather than the actual color will be more likely to choose the better example of that category—even if the hue they saw was not the best example of the category at test. The items around a boundary are more ambiguous and can be labeled in different ways based on context.

Hendrickson et al. (2012) use a category learning paradigm to investigate the label ambiguity hypothesis (Hanley and Roberson, 2011). They find that there is a pre-categorization asymmetry in addition to the enhanced effect after category learning. If the asymmetry exists prior to learning labels, label ambiguity alone cannot account for the asymmetry. They put forward an account based on unsupervised learning of clusters regardless of labeling.

Our experiments did not contain a classic categorical perception task since we only conducted within category trials. We also did not utilize a two alternative forced choice paradigm. However, the same/different task similarly requires participants to compare their memory for a stimulus to the test items. Rather than use all items as both target and foil, we had set targets with foils in both the typical and atypical direction. Therefore, each target hue was both a good exemplar (atypical trials) and a poor exemplar (typical trials) compared to the current foil. The research above would suggest that there should be enhanced performance on good exemplar, or atypical trials. This is the opposite of what we found. Sensitivity to differences decreased when the test hue was less typical of the category. The label ambiguity hypothesis cannot account for this result.

## Conclusion: memory, categorization and reasoning are intertwined

We examined the effect of active category labeling on hue memory creation. Memory even at 500 ms after initial perception is affected by categorical structure, regardless of active labeling. Given the short time scale and the reliable influence of category typicality, it seems safe to conclude that memory and categorization are inextricably intertwined in this task. While our experiments did not look at learning, labels are known to facilitate the learning of categories (Lupyan et al., 2007; Sloutsky and Fisher, 2012), which is considered to be a reasoning process. In so far as categorization is based on past experience, is ubiquitous in its influence on memory, and is developed at least in part through reasoning, the historically distinct topics of memory, categorization, and reasoning would appear to be comprised of common elements. As the topics continue to be considered together, the interrelations and underlying processes will become more clear.

### Conflict of interest statement

The authors declare that the research was conducted in the absence of any commercial or financial relationships that could be construed as a potential conflict of interest.

## References

[B1] Anderson M. C. Bjork R. A. Bjork E. L. (1994). Remembering can cause forgetting: retrieval dynamics in long-term memory. J. Exp. Psychol. Learn. Mem. Cogn. 20, 1063–1087 10.1037/0278-7393.20.5.1063 7931095

[B2] Berlin B. Kay P. (1969). Basic Color Terms: Their Universality and Evolution. Berkeley, CA: University of California Press

[B3] Blanco N. Gureckis T. M. (2013). Does category labeling lead to forgetting? Cogn. Process. 14, 73–79 10.1007/s10339-012-0530-4 23064883

[B4] Bukach C. M. Gauthier I. Tarr M. J. (2006). Beyond faces and modularity: the power of an expertise framework. Trends Cogn. Sci. 10, 159–166 10.1016/j.tics.2006.02.004 16516534

[B6] Cesario J. (2014). Priming, replication, and the hardest science. Perspect. Psychol. Sci. 9, 40–48 10.1177/1745691613513470 26173239

[B7] Churchland P. M. (1981). Eliminative materialism and the propositional attitudes. J. Philos. 78, 67–90 10.2307/2025900

[B8] Churchland P. M. (1988). Perceptual plasticity and theoretical neutrality: a reply to Jerry Fodor. Philos. Sci. 55, 167–187

[B9] City University. (2002). A Web-Based Colour Vision Test. Available online at: http://www.city.ac.uk/avrc/colourtest.html

[B10] Craik F. I. M. Tulving E. (1975). Depth of processing and the retention of words in episodic memory. J. Exp. Psychol. Gen. 104, 268–294 10.1037/0096-3445.104.3.26824156261

[B11] Farah M. J. (1996). Is face recognition “special”? evidence from neuropsychology. Behav. Brain Res. 76, 181–189 10.1016/0166-4328(95)00198-0 8734052

[B12] Gauthier I. Logothetis N. K. (2000). Is face recognition not so unique after all? Cogn. Neuropsychol. 17, 125–142 10.1080/026432900380535 20945176

[B13] Gauthier I. Skudlarski P. Gore J. C. Anderson A. W. (2000). Expertise for cars and birds recruits brain areas involved in face recognition. Nat. Neurosci. 3, 191–197 10.1038/72140 10649576

[B14] Goldstone R. L. (1994). Influences of categorization on perceptual discrimination. J. Exp. Psychol. Gen. 123, 178–200 8014612 10.1037//0096-3445.123.2.178

[B15] Goldstone R. L. (1998). Perceptual learning. Annu. Rev. Psychol. 49, 585–612 10.1146/annurev.psych.49.1.585 9496632

[B16] Goldstone R. L. Hendrickson A. T. (2010). Categorical perception. Wiley Interdiscip. Rev. Cogn. Sci. 1, 69–78 10.1002/wcs.26 26272840

[B17] Grill-Spector K. Kanwisher N. (2005). Visual recognition as soon as you know it is there, you know what it is. Psychol. Sci. 16, 152–160 10.1111/j.0956-7976.2005.00796.x 15686582

[B18] Hanley J. R. Roberson D. (2011). Categorical perception effects reflect differences in typicality on within-category trials. Psychon. Bull. Rev. 18, 355–363 10.3758/s13423-010-0043-z 21327385

[B18a] Harnad S. (1987). Categorical Perception: The Groundwork of Cognition. New York, NY: Cambridge University Press

[B19] Hayes B. K. Heit E. Rotello C. M. (2014). Memory, reasoning and categorization: parallels and common mechanisms. Front. Psychol. 5:529 10.3389/fpsyg.2014.00529 24987380 PMC4060413

[B20] Heit E. (1997). Knowledge and concept learning, in Knowledge, Concepts, and Categories, eds Lamberts K. Shanks D. (Hove: Psychology Press), 7–41

[B21] Heit E. Hayes B. K. (2005). Relations among categorization, induction, recognition, and similarity. J. Exp. Psychol. Gen. 134, 596–605 10.1037/0096-3445.134.4.596 16316295

[B22] Heit E. Rotello C. M. (2014). Traditional difference-score analyses of reasoning are flawed. Cognition 131, 75–91 10.1016/j.cognition.2013.12.003 24462712

[B23] Heit E. Rotello C. M. Hayes B. K. (2012). Relations between memory and reasoning, in Psychology of Learning and Motivation, ed Ross B. H (Academic Press), 57–101

[B24] Hendrickson A. T. Carvalho P. F. Goldstone R. L. (2012). Going to extremes: the influence of unsupervised categories on the mental carticaturization of faces and asymmetries in perceptual discrimination, in Proceedings of the Thirty-Fourth Annual Conference of the Cognitive Science Society, (Sapporo: Cognitive Science Society), 1662–1667

[B25] Hsieh P. J. Vul E. Kanwisher N. (2010). Recognition alters the spatial pattern of FMRI activation in early retinotopic cortex. J. Neurophysiol. 103, 1501–1507 10.1152/jn.00812.2009 20071627 PMC3257064

[B26] Huttenlocher J. Hedges L. V. Duncan S. (1991). Categories and particulars: prototype effects in estimating spatial location. Psychol. Rev. 98, 352–376 10.1037/0033-295X.98.3.352 1891523

[B27] Kanwisher N. McDermott J. Chun M. M. (1997). The fusiform face area: a module in human extrastriate cortex specialised for face perception. J. Neurosci. 17, 4302–4311 9151747 10.1523/JNEUROSCI.17-11-04302.1997 PMC6573547

[B28] Kikutani M. Roberson D. Hanley J. R. (2010). Categorical perception for unfamiliar faces: The effect of covert and overt face learning. Psychol. Sci. 21, 865–872 10.1177/0956797610371964 20483817

[B29] Levin D. T. Beale J. M. (2000). Categorical perception occurs in newly learned faces, other-race faces, and inverted faces. Percept. Psychophys. 62, 386–401 10.3758/BF03205558 10723217

[B30] Liberman A. M. Harris K. S. Hoffman H. S. Griffith B. C. (1957). The discrimination of speech sounds within and across phoneme boundaries. J. Exp. Psychol. 54, 358–368 10.1037/h0044417 13481283

[B31] Lupyan G. (2008). From chair to “chair:” A representational shift account of object labeling effects on memory. J. Exp. Psychol. Gen. 137, 348–369 10.1037/0096-3445.137.2.348 18473663

[B32] Lupyan G. (2012). Linguistically modulated perception and cognition: the label-feedback hypothesis. Front. Psychol. 3:54 10.3389/fpsyg.2012.00054 22408629 PMC3297074

[B33] Lupyan G. Rakison D. H. McClelland J. L. (2007). Language is not just for talking: labels facilitate learning of novel categories. Psychol. Sci. 18, 1077–1083 10.1111/j.1467-9280.2007.02028.x 18031415

[B34] Macmillan N. A. Creelman C. D. (2004). Detection Theory: A User's Guide, 2nd Edn. Mahwah, NJ: Lawrence Erlbaum Associates Inc

[B35] McKone E. Kanwisher N. Duchaine B. C. (2007). Can generic expertise explain special processing for faces? Trends Cogn. Sci. 11, 8–15 10.1016/j.tics.2006.11.002 17129746

[B36] Morris C. D. Bransford J. D. Franks J. J. (1977). Levels of processing versus transfer appropriate processing. J. Verb. Learn. Verb. Behav. 16, 519–533 10.1016/S0022-5371(77)80016-919882435

[B37] Nadel L. Hupbach A. Gomez R. Newman-Smith K. (2012). Memory formation, consolidation and transformation. Neurosci. Biobehav. Rev. 36, 1640–1645 10.1016/j.neubiorev.2012.03.001 22465050

[B38] Pylyshyn Z. (1999). Is vision continuous with cognition? The case for cognitive impenetrability of visual perception. Behav. Brain Sci. 22, 341–365 11301517 10.1017/s0140525x99002022

[B39] Regier T. Kay P. (2009). Language, thought, and color: whorf was half right. Trends Cogn. Sci. 13, 439–446 10.1016/j.tics.2009.07.001 19716754

[B40] Reyna V. F. Brainerd C. J. (1995). Fuzzy-trace theory: an interim synthesis. Learn. Individ. Differ. 7, 1–75 10.1016/1041-6080(95)90031-4

[B41] Rhodes G. Brennan S. Carey S. (1987). Identification and ratings of caricatures: implications for mental representations of faces. Cogn. Psychol. 19, 473–497 10.1016/0010-0285(87)90016-8 3677584

[B42] Richler J. J. Gauthier I. Palmeri T. J. (2011). Automaticity of basic-level categorization accounts for labeling effects in visual recognition memory. J. Exp. Psychol. Learn. Mem. Cogn. 37, 1579–1587 10.1037/a0024347 21767063

[B43] Richler J. J. Palmeri T. J. Gauthier I. (2013). How does using object names influence visual recognition memory? J. Mem. Lang. 68, 10–25 10.1016/j.jml.2012.09.001

[B44] Roberson D. Hanley J. R. Pak H. (2009). Thresholds for color discrimination in English and Korean speakers. Cognition 112, 482–487 10.1016/j.cognition.2009.06.008 19619872

[B45] Rosch E. (1975). The nature of mental codes for color categories. J. Exp. Psychol. Hum. Percept. Perform. 1, 303–322 10.1037/0096-1523.1.4.303

[B46] Simons D. J. (2000). Attentional capture and inattentional blindness. Trends Cogn. Sci. 4, 147–155 10.1016/S1364-6613(00)01455-8 10740279

[B47] Sloutsky V. M. (2003). The role of similarity in the development of categorization. Trends Cogn. Sci. 7, 246–251 10.1016/S1364-6613(03)00109-8 12804690

[B48] Sloutsky V. M. Fisher A. V. (2012). Linguistic labels: conceptual markers or object features? J. Exp. Child Psychol. 111, 65–86 10.1016/j.jecp.2011.07.007 21903223 PMC3185180

[B49] Snodgrass J. G. Corwin J. (1988). Pragmatics of measuring recognition memory: applications to dementia and amnesia. J. Exp. Psychol. Gen. 117, 34–49 2966230 10.1037//0096-3445.117.1.34

[B50] Stanislaw H. Todorov N. (1999). Calculation of signal detection theory measures. Behav. Res. Methods Instrum. Comput. 31, 137–149 10.3758/BF03207704 10495845

[B51] Sturges J. Whitfield T. W. A. (1995). Locating basic colours in the munsell space. Color Res. Appl. 20, 364–376 10.1002/col.5080200605

[B52] Toveé M. J. (1998). Is face processing special? Neuron 21, 1239–1242 10.1016/S0896-6273(00)80644-3 9883718

[B53] Whorf B. L. (1956). Language, Thought and Reality. Cambridge, MA: MIT Press

[B54] Winawer J. Witthoft N. Frank M. C. Wu L. Wade A. R. Boroditsky L. (2007). Russian blues reveal effects of language on color discrimination. Proc. Natl. Acad. Sci. U.S.A. 104, 7780–7785 10.1073/pnas.0701644104 17470790 PMC1876524

