# Supplementary material for: Representational shifts made visible: movement away from the prototype in memory for hue
Source: Front Psychol. 2014 Jul 31;5:796. doi: 10.3389/fpsyg.2014.00796 (PMC4117181; doi:10.3389/fpsyg.2014.00796)
Supplement: Supplementary file 1 [file Presentation1.PDF]

Appendixes

Appendix A. Hue calculations.

Hue Coordinates in LCH and Lab Color Spaces

Hue		L*	C	H	L*	a	b
g1--		42.842	42.909	135	43	-30	30
g1-	g2--	42.842	42.909	139	43	-32	28
**g1**	g2-	42.842	42.909	143	43	-34	26
g1+	**g2**	42.842	42.909	147	43	-36	23
g1++	g2+	42.842	42.909	151	43	-38	21
	g2++	42.842	42.909	155	43	-39	18
Focal Green		*42.842*	*42.909*	*157.039*	*43*	*-40*	*17*
g3++		42.842	42.909	159	43	-40	15
g3+	g4++	42.842	42.909	163	43	-41	13
**g3**	g4+	42.842	42.909	167	43	-42	10
g3-	**g4**	42.842	42.909	171	43	-42	8
g3--	g4-	42.842	42.909	175	43	-43	4
	g4--	42.842	42.909	179	43	-43	1
r1--		41.221	79.347	14	41	77	19
r1-	r2--	41.221	79.347	18	41	75	25
**r1**	r2-	41.221	79.347	22	41	73	30
r1+	**r2**	41.221	79.347	26	41	71	35
r1++	r2+	41.221	79.347	30	41	69	40
	r2++	41.221	79.347	34	41	66	45
Focal Red		*41.221*	*79.347*	*36.143*	*41*	*64*	*47*
r3++		41.221	79.347	38	41	62	49
r3+	r4++	41.221	79.347	42	41	59	53
**r3**	r4+	41.221	79.347	46	41	55	57
r3-	**r4**	41.221	79.347	50	41	51	61
r3--	r4-	41.221	79.347	54	41	46	64
	r4--	41.221	79.347	58	41	42	67

Note: The bold hues were shown at study. The focal hues are for reference; Participants did not see the focal hues.

Appendix B. Hit Rates and False Alarm Rates.

Hit and False Alarm Rates by Experiment and Judgment Conditions

		Hue Type				
Experiment	Condition	-2	-1	Target	1	2
1a	Category	0.638	0.770	0.805	0.730	0.518
	Preference	0.693	0.770	0.811	0.698	0.573
1b	Category	0.660	0.791	0.816	0.750	0.589
	Preference	0.636	0.801	0.827	0.731	0.615
2a	Category	0.646	0.782	0.743	0.655	0.490
	Preference	0.600	0.739	0.715	0.624	0.491
2b	Category	0.574	0.618	0.729	0.597	0.511
	Preference	0.576	0.651	0.662	0.595	0.482

Note: All numbers are proportions of hits to misses or false alarms to correct rejections. The target column represents the hit rate while the other columns are the matched lures. Sign denotes towards (+) or away (-) from the prototype and number (1, 2) denotes steps distant from the original hue.
